# Caffeine and NAD^+^ Improve Motor Neural Integrity of Dissociated Wobbler Cells In Vitro

**DOI:** 10.3390/antiox9060460

**Published:** 2020-05-27

**Authors:** Mareike Zwilling, Carsten Theiss, Veronika Matschke

**Affiliations:** Department of Cytology, Institute of Anatomy, Medical Faculty, Ruhr University Bochum, D-44801 Bochum, Germany; m.zwilling@online.de (M.Z.); carsten.theiss@rub.de (C.T.)

**Keywords:** amyotrophic lateral sclerosis, ALS, motor neuron disease, neuroprotection, ROS, degeneration, NAD^+^ precursor, Nmnat2

## Abstract

Amyotrophic lateral sclerosis (ALS) is a common degenerative disease of the central nervous system concerning a progressive loss of upper and lower motor neurons. While 5%–10% of patients are diagnosed with the inherited form of the disease, the vast majority of patients suffer from the less characterized sporadic form of ALS (sALS). As the wobbler mouse and the ALS show striking similarities in view of phenotypical attributes, the mouse is rated as an animal model for the disease. Recent investigations show the importance of nicotinamide adenine dinucleotide (NAD^+^) and its producing enzyme nicotinic acid mononucleotide transferase 2 (Nmnat2) for neurodegeneration as well as for the preservation of health of the neuronal cells. Furthermore, it is newly determined that these molecules show significant downregulations in the spinal cord of wobbler mice in the stable phase of disease development. Here, we were able to prove a positive benefit on affected motor neurons from an additional NAD^+^ supply as well as an increase in the Nmnat2 level through caffeine treatment in cells in vitro. In addition, first assumptions about the importance of endogenous and exogenous factors that have an influence on the wellbeing of motor nerve cells in the model of ALS can be considered.

## 1. Introduction

Amyotrophic lateral sclerosis (ALS) is the most common adult onset motor neuron disease. It is characterized by a degeneration of motor neurons in the brain and in the spinal cord [1]. Thus, the clinical phenotype involves weakness and atrophy as well as hyperreflexia and increased muscle tone [2]. While about 10% of the ALS cases represent the familial form, the disease occurs primarily sporadic [3]. Since there is no effective therapy yet, the average survival time after initial diagnosis is around 2–4 years [4]. Riluzole, a primarily, although not exclusively, anti-excitotoxic drug, and edaravone, an antioxidant, have only a minor beneficial impact on the course of the disease in a limited population of patients. The results on ropinirole, a dopamine receptor agonist, on ALS-derived iPSCs as a novel drug are not yet complete but auspicious [5].

The pathomechanisms within the ALS include various processes in the motor neurons such as protein aggregation, mitochondrial disruption and oxidative stress [6], as well as abnormal interactions with neighboring cells such as microglia and astrocytes [7,8]. Under pathological conditions, microglia cause secretion of pro-inflammatory mediators, and thus, local neuroinflammation leading to differential gene expression [7]. This can lead to an increased expression of reactive oxygen species (ROS)-producing molecules. Astrocytes can effect motor neurons by induced glutamatergic excitotoxicity [8].

Since the sporadic form affects quite a larger number of patients and is also much less understood than familial cases of the disease, the wobbler mouse as an animal model for the sALS is of special interest. The causal autosomal recessive gene defect in the vacuolar protein sorting-associated protein (*Vps54)* gene on chromosome 11 occurred spontaneously in the C57BL/Fa mouse strain [9]. *Vps54* is a gene locus encoding a part of the Golgi-associated retrograde protein (GARP) complex, which is important for the retrograde vesicle transport. A loss-of-function mutation in the *Vps54* gene leads to a disturbed vesicular transport, endosomal accumulation and at least protein aggregation, which finally induces death of the affected upper and lower motor neurons [10,11,12]. Protein aggregation, among others, also primarily characterizes the degenerative human ALS disease [13]. Besides, an activation of microglia and astrocytes as signs of inflammatory processes also plays an important role, both in the animal model as well as in human patients [7,8,14]. Nevertheless, the resemblance to the human ALS does also concern phenotypical aspects characterizing the wobbler mouse [15]. In wobbler mice, the progress of the disease can be described by means of various defined time points. Starting with the preclinical stage (p0) persisting about three weeks after birth characterized by a lack of phenotypical differences among the genotypes, disease development proceeds with the so-called evolutionary stage (p20). This stage involves the development of the clinical symptoms such as a wobbly gait, head tremor and muscle atrophy. Finally, the stabilized clinical phase (p40), characterized by sustained symptoms and an arrested progression of degeneration, follows [16,17].

Recent studies particularly reveal the importance of oxidative stress for the pathogenesis, both in wobbler mice and ALS patients [4,18]. The level of ROS as a marker for oxidative stress is found to be significantly elevated in the stabilized clinical phase (p40) of wobbler spinal cord [18]. It has been reported that ROS itself causes pathological processes such as the oxidation of DNA, protein and fatty acids leading to cell death [19]. Nicotinamide adenine dinucleotide (NAD^+^) plays a fundamental role in the process of ROS detoxification and moreover, in energy metabolism and mitochondrial function [20,21]. Furthermore, recent discoveries of NAD^+^ acting as a protector against PARP-1-mediated neuronal death and excitotoxicity-induced axonal degeneration expand the importance of NAD^+^ for further investigations on degenerative diseases [22,23]. The biosynthesis of NAD^+^ is mediated by the enzyme Nicotinic acid mononucleotide transferase (Nmnat). Three forms of this enzyme, Nmant1, Nmnat2, and Nmnat3, have different subcellular localizations [24]. Nmnat2 is the main isoform in mammalian brains localized in the cytosol and Golgi [24,25]. Previous studies show a positive impact of high Nmnat2 levels on neuronal health by a delay of axonal degeneration [26]. Nmnat2 depletion on the other hand seems to be sufficient to induce spontaneous degeneration [27]. The tight connection between Nmnat2, NAD^+^ and the degeneration of cells in a context of DNA damage suggests a potential relation to the tumor suppressor p53. It is reported that in an event of DNA damage, NAD^+^ is basically upregulated by Nmnat2 and p53 [28]. Concerning the wobbler disease, a downregulation of the Nmnat2 level in the clinical phase could be demonstrated, while p53 expression was evidently increased [18,29]. Apparently, a potential DNA damage due to increased oxidative stress [30,31] in wobbler motor neurons cannot be addressed in a physiological way, but presumably only by the p53 pathway. The missing elevation of Nmnat2 and the sole elevation of p53 might then lead to both downregulated NAD^+^ levels and motor neuronal death [28,32].

Caffeine is known for its antioxidant and neuroprotective properties. It protects cells against cell damage and is capable of reducing the risk of idiopathic neurodegenerative Parkinson’s disease [33,34]. As an antioxidant, caffeine is potent to reduce the level of biomarkers of oxidative stress [34] and achieves a reduction in oxidative stress-induced DNA-damage in rat lungs [35]. The antioxidative capability of caffeine can be explained by its scavenging effects on free radicals such as hydroxyl radicals [36] and the inhibition of peroxidation induced by these free radicals [37]. In addition to these antioxidative properties, caffeine was detected to cause an increase in the Nmnat2 level in neuronal cells [25]. Thus, the addition of caffeine to our motor neuronal feeding medium appears to be capable of normalizing Nmnat2 levels in wobbler motor neurons in a deficiency situation [18].

Since the NAD^+^ level was proved to be reduced in the cervical spinal cord of wobbler mice at p40 due to a significant downregulation of Nmnat2 [18], we aimed to investigate the impact of a NAD^+^ enhancement such as an increase in the Nmnat2 expression in motor neuronal cells of wobbler mice on their neurite lengths representative for neuronal health [38].

## 2. Materials and Methods

### 2.1. Animals

All procedures were conducted under established standards of the German federal state of North Rhine Westphalia, in accordance with the European Communities Council Directive 2010/63/EU on the protection of animals used for scientific purposes. Breeding and genotyping of mice was performed as previously described [17]. We used homozygous wildtype (WT) and wobbler (WR) mice of a C57BL/Fa mouse strain [39]. For all experimental settings for each genotype, and for each developmental stage, three separate cultures were performed using 2–3 WT and 2–3 WR animals at a time. Homozygous wildtype and wobbler as well as heterozygous spinal cord tissue were collected at two different stages (evolutionary phase (p20), and stable clinical phase (p40)) and used for further experiments.

### 2.2. Generation of C2C12 Myocyte-Conditioned Medium

The generation of C2C12 myocyte-conditioned medium was performed like previously described by Montoya-Gacharna et al. 2012 [40]. In brief, C2C12 myocytes were cultured in a culture bottle containing proliferation medium (20% FCS, 1% Pen/Strep, 4.5 g/L D-glucose in Dulbecco’s modified eagle medium (DMEM)) until the settlement attained a confluence of 30%. Medium was then exchanged for differentiation medium (20% horse serum, 1% Pen/Strep, 4.5 g/L D-glucose in DMEM) after washing once with motor neuron basal medium (MBM; 0.5 mM glutamine, 100 U/mL Pen/Strep, 2% B27 supplement in Neurobasal A) and twice with differentiation medium. After 3 days, differentiation medium was replaced by MBM and the supernatant was collected after another 2 days. After sterile filtration, the medium was stored at −80 °C.

### 2.3. Motor Neuron Enriched Dissociated Cell Culture of the Ventral Horn

To study the growth and regeneration of motor neurons ex vivo, a dissociated culture of the ventral horn was performed according to Montoya-Gacharna et al. 2012 [40] and Brewer and Torricelli 2007 [41].

In brief, the spinal cord of WT and WR mice was isolated in its entirety. After removing the meninges, the ventral horn was separated from the dorsal horn. The dorsal horn was discarded, and each ventral horn was cut into pieces of 500 µm. The tissue pieces were digested in enzyme digestion solution containing 0.02% DNase and 36 U/mL Papain in an isolation medium (0.5 mM GlutaMax, 100 U/mL Pen/Strep, 2% B27 supplement in Hibernate A) on a shaker for 5 min at room temperature and for an additional 25 min at 30 °C. After centrifugation at 1040× *g* for 5 min at room temperature, the supernatant was discarded. After trituration of the tissue, an OptiPrep density gradient centrifugation according to Brewer und Torricelli 2007 [41] was performed, to separate spinal cord cells from detritus and erythrocytes. Layer 2 and 3 according to Brewer and Torricelli 2007 [41] were collected in an isolation medium and centrifuged at 244× *g* for 6 min at 10 °C. The pellet was resuspended in 1 mL motor neuron feeding medium (MFM; MBM supplemented with 30% C2C12 myocyte-conditioned medium, 125 mM cyclic adenosine monophosphate (cAMP), 1 ng/mL brain-derived neurotrophic factor (BDNF), 0.1 ng/mL glial cell-derived neurotrophic factor (GDNF)). The cells were counted and seeded with a density of 70,000 cells/well on a 50 µg/mL poly-d-lysine (PDL)-laminated 12 mm glass slide. The medium was exchanged on the subsequent day of the preparation and afterwards, every second day.

For investigations concerning the influence of NAD^+^ and an increased Nmnat2 level on the growth of motor neurons, the medium was completed with 10 mM NAD^+^ or 10 µM caffeine according to previous studies [22,25]. The motor neuronal feeding medium was replaced by the extended medium from the day of preparation until the day of fixation. Cells were cultured for 7, 10 or 14 days.

### 2.4. Immunofluorescence Staining

To take measurements of the motor neuronal neurites, the cultured cells were stained by using the immunofluorescence method. The cells were fixated with 4% paraformaldehyde (PFA) for 20 min and washed 3 times (2′/wash) with phosphate-buffered saline (PBS). After permeabilization with 0.3% triton in PBS for 15 min, the cells were again washed 3 times (5′/wash) with PBS. The unspecific binding sites were blocked with goat serum (1:50 in PBS) for 30 min at room temperature and then shortly washed with PBS. For detection of the neurites, a rabbit neurofilament-M (NFM) antibody (1:500; #AB1987, Millipore, Burlington, MA, USA) was used at 4 °C overnight. The cultures were washed 3 times (7′/wash) with PBS (#P4417, Sigma-Aldrich, Louis, MO, USA) and subsequently incubated with the secondary antibody (1:1000; anti-rabbit Alexa Flour 488; #A21206, Thermo Fisher Scientific, Waltham, MA, USA) for 2 h at room temperature. After 3 times of washing (7′/wash) with PBS, the nuclear staining of the dissociated cells was performed with 4′,6-diamidino-2-phenylindole (DAPI; 1:1000; #D9542, Sigma-Aldrich, Louis, MO, USA) for 15 min at room temperature. Finally, the cells were again washed 3 times (6′/wash) before covering the plates with Fluoroshield (#F6937, Sigma-Aldrich, Louis, MO, USA) mounting medium.

### 2.5. Morphometric Analysis of Neurite Length

**Figure 1:**
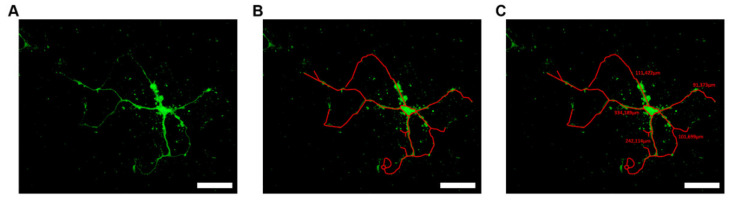
Exemplary measurement of motor neuronal neurite lengths. (**A**) Motor neuronal cell cultures were cultivated for an appropriate duration, fixed and stained with antibodies (Neurofilament M—green; Nucleus DAPI—blue). (**B**) Cultures were imaged with the aid of a confocal laser scanning microscope in combination with a 20× objective. (**C**) Measurement and summation of neurite length was achieved with the aid of the ZEN 2.3 lite measurement tool. Scale bar = 50 µm.

To investigate the effect of NAD^+^ and caffeine on neurite length of motor neurons of WT and WR mice, cultures were imaged with the aid of a confocal laser scanning microscope (LSM 800, Carl Zeiss Microscopy GmbH, Jena, Germany) equipped with the respective filter sets in combination with a 20× objective (Plan-Apochromat 20×/0.8, Carl Zeiss Microscopy GmbH, Jena, Germany). Measurement and summation of neurite length was achieved with the aid of the ZEN 2.3 lite measurement tool (Carl Zeiss Microscopy GmbH, Jena, Germany) (Figure 1). At least 10 motor neuronal cells were measured for each condition and each timepoint of three different cell isolations.

### 2.6. RNA Isolation, Reverse Transcription and Quantitative PCR

In order to investigate whether the use of caffeine caused overexpression of Nmnat2 in dissociated cells of the spinal cord, the total RNA of the cultures was isolated after cultivation and incubation with 10 µM caffeine for 7 days using the NucleoSpin miRNAKit (#740971, Macherey-Nagel, Düren, Germany) according to the manufacturer’s protocol. cDNA synthesis for mRNA amplification was performed using qScript cDNA Synthesis Kit (#95048, Quanta, Beverly, MA, USA). The quantitative real-time PCR (qPCR) was performed using GoTag qPCR Master Mix (#A6001, Promega, Madison, WI, USA) on a CFX96 Real-Time PCR Detection System (Bio-Rad, Hercules, CA, USA). The housekeeping gene glyceraldehyde 3-phosphate dehydrogenase (*GAPDH*) was used for normalization. Specific primer for *GAPDH* were sense, 5′-GGA GAA ACC TGC CAA GTA TGA-3′, and antisense, 5′-TCC TCA GTG TAG CCC AAG A-3′. Primer sequences for detection of *Nmnat2* were sense, 5′-AGA ACA CCC AGC CCA TTT AC-3′, and antisense, 5′-GAG GCT TTC TCC CAC CTT TC-3′. Expression levels were analyzed in three independent qPCR runs in triplicate and normalized with the appropriate housekeeper. Furthermore, the obtained Ct-values were analyzed using the 2^−ΔΔCt^ method [42].

### 2.7. Statistical Analysis

Statistical analyses of the data were performed with Prism 6.0 (GraphPad Inc., La Jolla, CA, USA). Data represent mean values of at least three independent experiments ± standard error of the mean (SEM). For the heatmap, the measured neurite lengths at each stage (p20 and p40) were normalized to the mean value of the appropriate wildtype value at 7 days in vitro (DIV) and mean percentage values were displayed. Data were tested for significance using two-way ANOVA with a Sidak post hoc test. Results with *p* < 0.05 were considered statistically significant.

## 3. Results

In order to investigate whether the degeneration of motor neurons of wobbler mice is caused by endogenous or exogenous factors, in this study, the development of these cells was assessed in vitro by examining the neurite lengths. The neurite length is closely linked to the level of cell health [38]. Due to the isolation of the spinal cord and the isolated cultivation of the cells in vitro, motor neurons do not experience any influences from attached cells, so that predominantly endogenous factors determine the development of the like. Motor neuronal cells of mice of two developmental stages (p20, p40) were analyzed after 7, 10, and 14 days in vitro (DIV). To objectify the morphology, and thus, the health of the wildtype and wobbler motor neurons in vitro, the neurite lengths were measured with the aid of the ZEN 2.3 lite software (Carl Zeiss Microscopy GmbH, Jena, Germany).

### 3.1. Altered Morphology and Development of Wobbler Motor Neurons In Vitro

**Figure 2:**
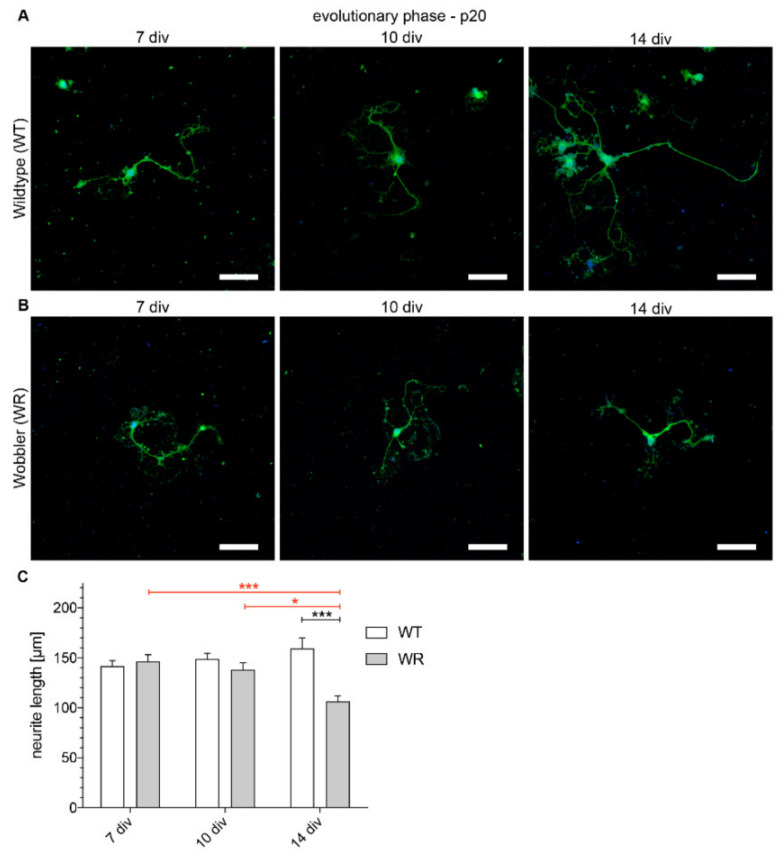
Neurites from wobbler motor neurons of the evolutionary phase (p20) are significantly shorter than wildtype cells after 14 DIV. (**A**) Exemplary images of dissociated motor neuronal cells of wildtype spinal cord of the evolutionary phase (p20) after 7, 10 and 14 DIV stained with anti-NFM antibody (green) and DAPI (nucleus, blue). (**B**) Exemplary images of dissociated motor neuronal cells of wobbler spinal cord of the evolutionary phase (p20) after 7, 10 and 14 DIV stained with anti-NFM antibody (green) and DAPI (nucleus, blue). (**C**) Neurite lengths of p20 motor neurons after 7, 10 and, 14 DIV. Constant uptrend of wildtype (WT) neurite lengths while wobbler (WR) neurites show a significantly loss in length over time in a sense of degeneration. Different development of the genotypes up to a significant difference in neurite length after 14 DIV can be observed. Data are provided as means ± SEM. Data were tested for significance using two-way ANOVA with a Sidak post hoc test. Significant differences are indicated by * *p* < 0.05, *** *p* < 0.001. *N* = 56–150. Scale bar = 50 µm.

Comparing the absolute lengths and the growth of wildtype and wobbler neurites of motor neurons isolated from 20- or 40-days old mice, striking differences emerge. The neurite length of motor neurons of wildtype and wobbler mice isolated from evolutionary stage animals (p20) shows no differences after 7 and 10 DIV. Only after 14 DIV, a significant difference in the length of neurites between the genotypes can be detected (Figure 2). Regarding the individual development of the genotypes in the course of 14 DIV, there is a constant but not significant growth concerning the wildtype neurite lengths (Figure 2C). Wobbler motor neurons show a steadily decreasing trend in neurite length with a significant difference between the lengths after 7 and 14 DIV and 10 and 14 DIV (Figure 2C).

**Figure 3:**
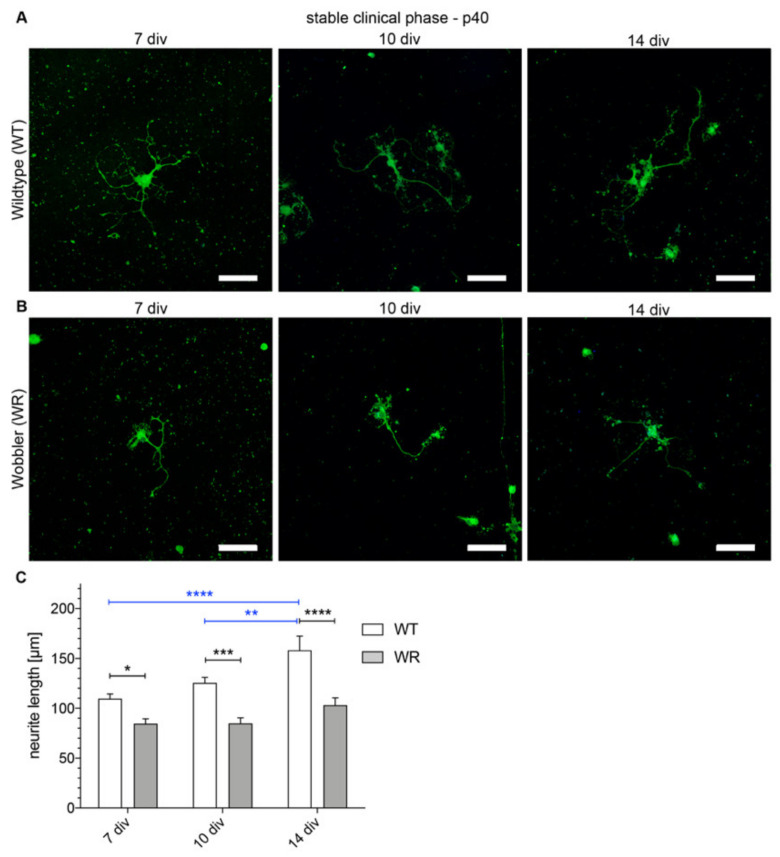
Neurites from wobbler motor neurons of the stable clinical phase (p40) are significantly shorter than wildtype cells at any measured time point. (**A**) Exemplary images of dissociated motor neuronal cells of wildtype spinal cord of the stable clinical phase (p40) after 7, 10 and 14 DIV stained with anti-NFM antibody (green) and DAPI (nucleus, blue). (**B**) Exemplary images of dissociated motor neuronal cells of wobbler spinal cord of the stable clinical phase (p40) after 7, 10 and 14 DIV stained with anti-NFM antibody (green) and DAPI (nucleus, blue). (**C**) Neurite lengths of p40 motor neurons after 7, 10 and, 14 DIV. Wildtype neurites show a constant significant growth in neurite length over time. Wobbler motor neurons achieve only a non-significant spreading of neurites over a time of 14 DIV. Neurite lengths of the two genotypes significantly differ at each timepoint. Data are provided as means ± SEM. Data were tested for significance using two-way ANOVA with a Sidak post hoc test. Significant differences are indicated by * *p* < 0.05, ** *p* < 0.01, *** *p* < 0.001, **** *p* < 0.0001. *N* = 77–147. Scale bar = 50 µm.

The investigation of wildtype and wobbler neurite lengths of motor neurons isolated from clinical stage animals (p40) show different results compared to the cells of the evolutionary stage (p20; Figure 3). Wobbler neurites reveal a trend to grow in length while wildtype neurites show a significant growth over time (Figure 3C). Significant differences between the lengths of wildtype neurites can be seen after 7 and 14 DIV and 10 and 14 DIV (Figure 3C). The neurites of wobbler motor neurons thus seem to extend much less, since no significances regarding the length comparisons within this genotype can be observed. This can also be underscored by the direct comparison between wildtype and wobbler. There is a significant difference between the length of wildtype and wobbler neurite lengths at all timepoints which points out the distinction of the growth behavior of both genotypes (Figure 3C).

### 3.2. NAD^+^ as Well as Caffeine Provides Beneficial Effects on Motor Neuronal Integrity In Vitro

As the coenzyme NAD^+^ was found to be significantly downregulated in the spinal cord of wobbler animals [18], we aimed to investigate the effect of a NAD^+^ boost on the motor neuronal development. The investigations were equally performed with two developmental stages (p20, p40). Neurite lengths were measured after 7, 10 and 14 DIV.

**Figure 4:**
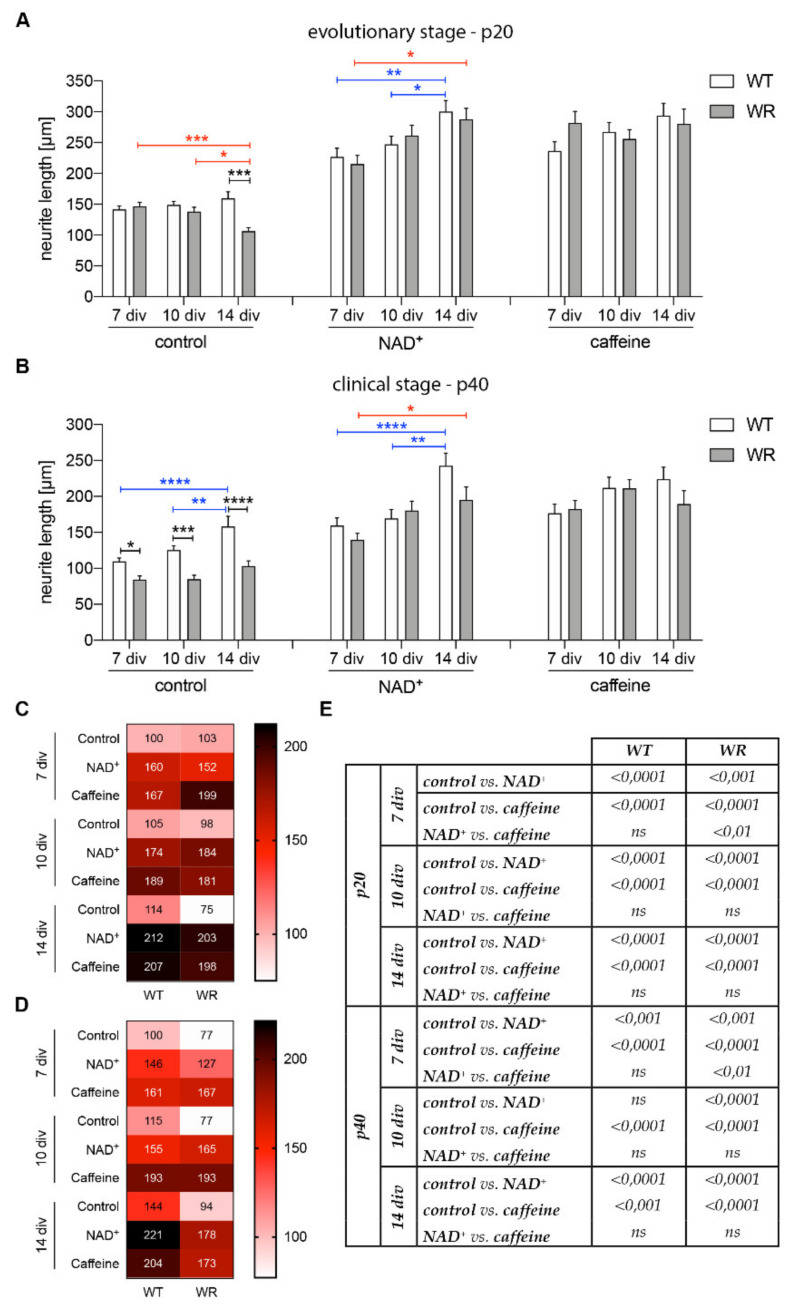
Increase in the NAD^+^ level in the culture medium as well as an intervention in the NAD^+^ metabolism through caffeine treatment improves motor neural integrity in vitro. (**A**) Measurement of neurite lengths of non-treated, NAD^+^ (10 mM) or caffeine (10 µM) supplemented wildtype and wobbler motor neurons at the evolutionary stage (p20). NAD^+^ treated WT as well as WR motor neurons show a significant gain in neurite length over time (*N* = 38–76). Diseased cells do not significantly differ in neurite length from control cells at all timepoints, which outlines the positive effect of an NAD^+^ enhancement on the degenerative development of wobbler motor neurons. Caffeine supplementation leads to a slight non-significant uptrend in neurite length of WT motor neurons in the p20 trial (*N* = 38–64). WR cells show a stagnant process over time. No significant differences can be observed. Data are provided as means ± SEM. (**B**) Investigation of p40 motor neurons in vitro treated with both NAD^+^ (*N* = 28–94) or caffeine (*N* = 20–89) reveal a development of neurite lengths similar to p20 motor neurons. Data are provided as means ± SEM. (**C**) Heatmap based on the change of neurite lengths depending on the treatment of p20 wildtype and wobbler motor neurons in vitro. The measured neurite lengths were normalized to the mean value of the wildtype at 7 DIV and percentage values were displayed. Both NAD^+^ treatment as well as caffeine addition significantly lead to a growth in neurite length compared to the neurite length of control at each time point. The downward trend in neurite length regarding non-treated motor neurons changes to a constant upward trend by the NAD^+^ treatment and a stagnant process by the caffeine treatment can be observed. (**D**) Heatmap based on the change of neurite lengths depending on the treatment of p40 wildtype and wobbler motor neurons in vitro. The measured neurite lengths were normalized to the mean value of the wildtype at 7 DIV and percentage values were displayed. Significant extension of wildtype and wobbler neurite lengths after treatment with NAD^+^ and caffeine as against studies without treatment at all steps of fixation. (**E**) *p*-values of multiple comparison of the effect of different culturing conditions on the length of motor neuronal neurites at different cultivation times. Data were tested for significance using two-way ANOVA with a Sidak post hoc test. Significant differences are indicated by * *p* < 0.05, ** *p* < 0.01, *** *p* < 0.001, **** *p* < 0.0001. ns = not significant.

A common increase in motor neuronal neurite length of both wildtype and wobbler genotype isolated from evolutionary stage animals (p20) after NAD^+^ treatment compared to control conditions can be observed (Figure 4A,B). The neurite length, depending on the NAD^+^ treatment and the cultivation time, shows a significant difference between wildtype neurites cultivated for 7 and 14 DIV and between a cultivation time of 10 and 14 days (Figure 4A). A significant growth of neurites from wobbler mice between 7 and 14 DIV is obvious (Figure 4A). Comparing the genotypes at each time point, there is no significant disparity between the neurite lengths in NAD^+^ treated motor neurons detectable (Figure 4A). Comparing the neurite length after NAD^+^ treatment with control conditions, a highly significant increase in neurite length was observed in wildtype cells at each examined time point (Figure 4B,E). A significant increase in length was also observed in wobbler cells at all time points after incubation with NAD^+^, although the effect is slightly less significant after 7 DIV (Figure 4B,E).

The neurite length of motor neurons of wildtype and wobbler mice isolated from clinical stage animals (p40) shows similar findings after NAD^+^ treatment (Figure 4C,D). Wobbler as well as wildtype neurites undergoing a NAD^+^ treatment significantly gain in length over the time of 14 DIV (Figure 4C). Again, we found significant differences between wildtype neurites after 7 and 14 DIV and 10 and 14 DIV. Wobbler neurites of motor neurons significantly differ in length after 7 and 14 DIV. We did not find any striking variations between the genotypes at the particular time points (Figure 4C). Comparing the neurite length after NAD^+^ treatment with control conditions, a significant increase in neurite length was observed in wildtype cells at 7 and 14 DIV (Figure 4D,E). While a significant increase in length was observed in wobbler cells at all three time points after incubation with NAD^+^, the effect was slightly less significant after 7 DIV (Figure 4D,E).

Since the Nmnat2 level was proven to show a significant downregulation in the spinal cord of wobbler mice [18], we aimed to study the effect of an elevation of the Nmnat2 level on motor neuronal integrity in vitro. Since Ali and coworkers have shown that caffeine is able to increase the Nmnat2 level in cortical neurons in vitro [25], we used this compound as treatment of our cell culture.

Within the motor neuronal cells of wildtype mice isolated from evolutionary stage animals (p20), a continuous upward trend in neurite length after caffeine treatment can be observed (Figure 4A,B). Motor neuronal cells of wobbler mice isolated from p20 show a stagnant process over time. In this regard, we could not find any significances especially regarding the comparison between the genotypes at each investigated time point (Figure 4A). Comparing the neurite length after caffeine treatment with control conditions, a highly significant increase in neurite length was observed in wildtype as well as in wobbler cells at each examined time point (Figure 4B,E). The neurite lengths of motor neurons of wildtype and wobbler mice isolated from clinical stage animals (p40) show similar findings after the addition of caffeine (Figure 4C,D). Comparing the neurite length after caffeine treatment with control conditions, a significant increase in length was observed in wildtype cells at all time points after incubation with NAD^+^, although the effect is slightly less significant after 14 DIV (Figure 4D,E), whereas a highly significant increase in length was observed in wobbler cells at all three time points (Figure 4D,E).

Interestingly, except for two stages of wobbler cells (p20, 7 DIV and p40, 7 DIV), no difference in the effect between NAD^+^ and caffeine treatment was found (Figure 4E). For both exceptions, treatment with caffeine showed a significantly better result regarding the length of neurites of wobbler motor neurons.

### 3.3. Caffeine Increases Nmnat2 Level in Dissociated Cell Cultures of the Spinal Cord

To prove the elevation of Nmnat2 mRNA level in dissociated cell cultures of the spinal cord after addition of caffeine, we cultured isolated cells of evolutionary (p20) and stable clinical (p40) stage for 7 DIV and performed quantitative PCR.

**Figure 5:**
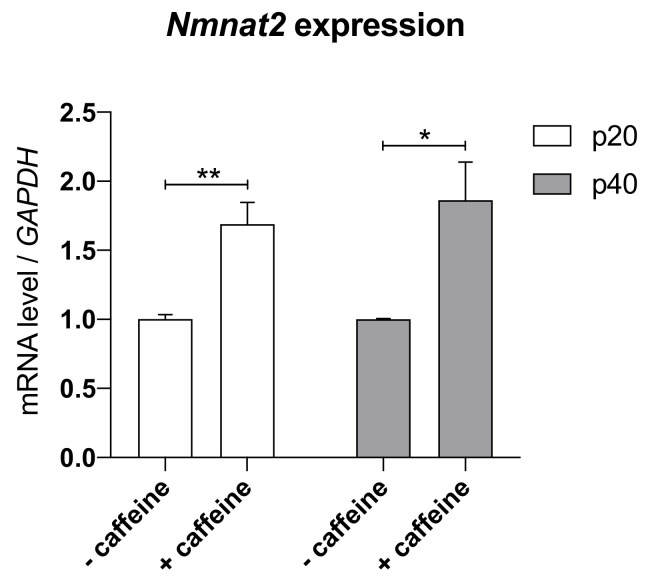
Increased Nmnat2 mRNA expression in motor neuron enriched dissociated cell cultures of the ventral horn after caffeine treatment. The qPCR was performed using three samples for each treatment and stage. A significant upregulation of *Nmnat2* mRNA could be detected at both stages after caffeine treatment. For relative quantification of *Nmnat2* expression, the 2^−∆∆Ct^ method was conducted using the housekeeping gene *GAPDH* for normalization. Data are provided as means ± SEM. Data were tested for significance using Student’s *t* test. Significant differences are indicated by *p < 0.05, **p < 0.01; *n* = 3.

A significant enhancement of the mRNA level of Nmnat2 in caffeine treated cultures in contrast to cells without any treatment in p20 and p40 cultures could be observed (Figure 5).

## 4. Discussion

In vitro studies open a new perspective on the development of diseased motor neurons and the influence of endogenous and exogenous factors triggering the molecular changes and degenerative course. While there was found to be various pathological phenomena within the wobbler mouse, such as an enlargement of endosomal vesicles [11], an impairment of the anterograde and retrograde axonal transport [43] or a mitochondrial dysfunction [44], there is still a lack of investigations regarding the beginning of the degenerative causal chain. The *Vps54* gene defect is not yet sufficient to explain all cellular pathologies within the wobbler motor neurons [10,45]. By isolating the cells of the ventral horn from remaining tissues and compartments of the organism, we were able to create standardized conditions and independence from potential systemic triggers. The isolation leads to sole as well as reduce exposure to possible influences of neighboring cells within the ventral spinal cord and their emitted messenger substances [14,18,46,47]. In addition, the motor neurons might be subjected to their own endogenous factors being important for disease development.

Wobbler motor neurons from evolutionary stage (p20) show a significant loss of neurite lengths over a time of 14 DIV, while wildtype cells show a certain increase in neurite length over time. This different development leads to a significant difference in neurite lengths between the genotypes after 14 DIV. The development of p20 wobbler motor neurons ex vivo thus seems to be just like in an ordinary environment, since it is known that from p20 onwards, these cells start to degenerate in diseased mice [15]. This finding might be a first hint to an organism independent degeneration of concerned cells in the evolutionary stage of disease development.

By contrast, we found a continuous non-significant uptrend in neurite length of wobbler motor neurons from clinical stage (p40) in vitro. This suggests the assumption that cellular processes fundamentally differ between the developmental stages p20 and p40. Even though WR neurite lengths are significantly shorter than WT neurites apposite to the common degenerative processes within the animals, it can be hypothesized that in stable clinical phase the cells might have resources to regenerate in vitro, while in vivo circumstances affect the processes in a yet unknown negative way and inhibit any recovery of diseased motor neurons. These processes occur although the *Nmnat*2 mRNA level and NAD^+^ level are significantly reduced, especially in the p40 stage [18]. Beyond this, pro-inflammatory activities of microglial cells previously demonstrated in an in vivo experimental setup [48] might differ in an in vitro setting, so that they might exert less negative influences on the motor neurons.

NAD^+^ is primarily known as an essential coenzyme in energy metabolism, gene expression and DNA repair. Recently, NAD^+^ was connected to other processes such as neuroprotection and the reduction in oxidative stress [21,49] as well as aging and longevity [50]. The present study based on recent findings of a reduction in the NAD^+^ level in the spinal cord of p40 wobbler mice [18] investigates the effect of an NAD^+^ enhancement on the motor neuronal development in vitro.

At p20, wobbler motor neurons undergoing NAD^+^ treatment show a significant increase in neurite length over 14 DIV. The switch from a constant decrease in neurite length in studies without any treatment to an increase suggests a severe positive impact of NAD^+^ on neuronal cell integrity. A direct comparison of neurite lengths of non-treated wobbler motor neurons and NAD^+^ treated cells reveal significant differences at all examined time points, which underscores the effect of NAD^+^ on the diseased cells. At p40, NAD^+^ treated cells compared to non-treated wobbler motor neurons show a significant increase in neurite length. Significant differences between the genotypes at each time point are abolished in a NAD^+^ setting, pointing out the approximation of diseased cells to normal growth behavior. Comparing non-treated and NAD^+^ boosted motor neurons at all specific time points, significant differences emerge and reveal a significant benefit of diseased motor neurons.

The positive effect of an NAD^+^ enhancement underlines the importance of the reported NAD^+^ depletion on the motor neuronal degeneration within the wobbler model. Since the authors in [18] reported not only a decreased NAD^+^ level but also an increased ROS level in the clinical phase of disease development, mechanisms of the degenerative escape of p40 wobbler motor neurons by NAD^+^ boosting might primarily include NAD^+^ mediated ROS detoxification and the resulting lower level of oxidative stress [51]. Whereas the finding of a differential occurrence of ROS and NAD^+^ solely applies to p40 spinal cord, the described chaining does not seem to pertain to p20 studies. However, a significant increase in neurite lengths due to NAD^+^ treatment was also shown in the control. Regardless of a previous deficiency situation, the NAD^+^ overabundance thus also achieves an improvement of the motor neuronal integrity, which requires further explanations on the NAD^+^ effect. The direct neuroprotective function of NAD^+^ through various mechanisms such as a remediation of DNA repair activity [49], a restoration of ATP levels and a maintenance of membrane integrity [52] might be an important factor explaining positive effects of a NAD^+^ boost in a non-deficient situation.

As NAD^+^ metabolism was further described to be involved in the pathomechanisms of different neurodegenerative diseases, such as the Parkinson’s disease, Alzheimer’s disease and the retinal degenerative disease [53], scientists are currently exploring pharmacological ways of an NAD^+^ increasement yet. Three options are described so far: administration of NAD^+^ precursor, activation of NAD^+^ biosynthetic enzymes, and inhibition of NAD^+^ degradation [54]. NAD^+^ precursor are even described to have a miraculous benefit on the development of age-related and metabolic disorders in preclinical studies, which is meant to be further investigated in clinical trials [55]. The specific effect on the wobbler model, and accordingly, the human ALS disease, still needs to be examined but appears promising considering the current findings of this study.

Nmnat2 is an NAD^+^ synthesizing enzyme in the Preiss-handler and the salvage pathway from the precursors nicotinamide mononucleotide (NMN) and nicotinic acid (NA) by transferring adenosine monophosphate (AMP). Nmnat is, thus, an essential enzyme for two out of three pathways of the NAD^+^ generation. All pathways use different substrates wherefore the election of the synthesis methods may be dependent on both substrate availability and the level of required enzymes [56]. Since Nmnat proteins are, thus, the most essential generators of NAD^+^ and the mRNA level of Nmnat2 was found to be significantly downregulated in p40 wobbler spinal cord [18], further investigations were performed using caffeine as a dilating ingredient in the motor neuron, feeding medium to elevate the Nmnat2 level in motor neuronal cells.

At p20 and p40, measurements revealed a non-significant stagnation of neurite lengths of both wildtype and wobbler motor neurons undergoing caffeine treatment over a time of 14 DIV. No significance can be found concerning the difference between the genotypes at any time point both in p20 and p40 studies, which points out the convergence of wildtype and wobbler cell development. However, a significant increase in neurite length of wildtype and wobbler motor neurons through a Nmnat2 increasement at both developmental stages compared to control cells could be shown.

Again, a consistently beneficial influence of the extended medium on wobbler motor neurons can be seen after caffeine treatment. If it is assumed that an increase in Nmnat2 levels leads to an increase in NAD^+^ production, it could be concluded that the effects found are based on the same mechanisms as in previous NAD^+^ studies. Besides, the level of NAD^+^ precursor, like NMN, would be reduced through the enhanced turnover rate of Nmnat2. Since NMN was found to be toxic and to promote axon degeneration [57], a decreased NMN level could, thus, explain the displayed positive effects.

Furthermore, Nmnat2 was found to be required for Golgi structure. Nmnat2 depletion was proven to lead to a fragmentation of the Golgi apparatus in neurons. This itself leads to an activation of apoptotic pathways concerning an elevated activation of caspase 6 [58]. Since the Golgi apparatus was also described to play a fundamental role in the pathogenesis of the wobbler and human ALS disease in a sense of a structural and functional disorder [10,11,59], the connection between Nmnat2 and the Golgi apparatus appears relevant to explain Nmnat2 effects on wobbler motor neurons. Recently, the link between neuron degeneration in wobbler mice and the apoptotic pathways including caspase activity was investigated by the authors in [45]. Considering that caspase 3 and 6 were proven to show a coordinated activation in the apoptosis of cerebellar granule cells [60], the results within the mentioned study, such as an increased activeness of caspase 3, agree with previous assumptions [45].

However, the effect of a caffeine treatment is found, although not significant, to be less prominent after 14 DIV than the effect of a NAD^+^ treatment. These findings indeed raise the question why an increase in NAD^+^ can generate an evidently more severe impact on wobbler motor neurons than the increase in Nmnat2. Even though Nmnat2 is known to be directly associated with the coenzyme, the correlation between the two molecules is not yet defined in the sense of a linear relationship. This limits the previous explanations of the Nmnat2 effect via an NAD^+^ increase. Furthermore, the elevation of Nmnat2 in motor neurons in this study was performed indirectly through the addition of caffeine. The signaling cascade, through which caffeine leads to an increasement in the Nmnat2 level, includes the role as a phosphodiesterase inhibitor to reduce cAMP degradation, and thus, raise cAMP concentrations. cAMP itself then acts as a mediator to activate the cAMP-response element binding protein (CREB). CREB then regulates the transcription of Nmnat2 through a binding on the Nmnat2 promoter [25,61]. Due to the multiple indirections, a potential susceptibility to interference in the availability of substrates of all intermediate reactionary steps and an extended time until the aimed effect occurs can be assumed.

Nevertheless, the direct neuroprotective effect of Nmnat2 should be also mentioned as an important factor leading to a positive change in the development of diseased motor neurons. It is reported that an overexpression and stabilization of Nmnat2 can significantly protect neurons from Wallerian degeneration [27]. Nmnat2 thus could be identified as one critical axon survival factor, required to maintain regular axon integrity and capable to obtain injured axons at high doses [26].

## 5. Conclusions

In this study, we were able to survey the in vitro development of wobbler motor neurons in a dissociated culture of the ventral horn. Studies with motor neuronal cells out of p20 animals revealed an in vivo like cell development, while motor neurons out of p40 wobbler mice showed in vitro a slightly improved outcome compared to a physiological setting. Furthermore, it was shown that the addition of the coenzyme NAD^+^ to the motor neuronal culture medium achieves a benefit on motor neuronal development in vitro. Since it was found that Nmnat2 as an NAD^+^ producing enzyme also showed depleted levels in wobbler spinal cord as well, the effect of a Nmnat2 enhancement was analyzed with the aid of a caffeine treatment. In relation to the NAD^+^ studies, an alike but less concise positive effect was detected in this study when considering all three examined time points.

All in all, caffeine as well as NAD^+^ were both shown to have a positive impact on the in vitro development of motor neurons of wobbler mice, and thus, purport to be promising agents for further investigations into potential pharmacological treatments of both wobbler and ALS disease.
